# Foot placement control underlies stable locomotion across species

**DOI:** 10.1073/pnas.2413958122

**Published:** 2025-10-21

**Authors:** Antoine De Comite, Nidhi Seethapathi

**Affiliations:** ^a^McGovern Institute for Brain Research, Massachusetts Institute of Technology, Cambridge, MA 02139; ^b^Department of Brain and Cognitive Sciences, Massachusetts Institute of Technology, Cambridge, MA 02139; ^c^Department of Electrical Engineering and Computer Science, Massachusetts Institute of Technology, Cambridge, MA 02139

**Keywords:** motor control, stability, human, mouse, *Drosophila*

## Abstract

Animals navigate their environment stably without inefficient course corrections despite unavoidable errors. In humans, this stability is achieved by modulating the placement of the foot on each step such that recent errors are corrected. However, it is unknown whether animals with diverse nervous systems and body mechanics use such foot placement control; foot trajectories of many-legged animals are considered to be stereotypical velocity-driven patterns, as opposed to error-driven. Here, we put forth a unified “feedforward-feedback” control structure for stable locomotion that combines velocity-driven and body state error-driven foot placement. We provide empirical support for this control structure across flies, mice, and humans by mining their natural locomotor variability, finding that a competing control structure with purely velocity-driven foot placement is not supported by the data. This work finds shared behavioral signatures of foot placement control in flies, mice, and humans. We find that key characteristics of these signatures, such as their urgency and centralization, vary with neuromechanical embodiment across species. For example, more inherently stable multilegged animals exhibit less urgent control with a lower control magnitude and a slower correction timescale compared to humans. Furthermore, many-legged animals display modular, direction-, and leg-specific control signatures, whereas humans exhibit common signatures across both legs. Overall, our findings provide insight into stable locomotion across species, revealing how species with diverse neuromechanics achieve a shared functional goal: foot placement control.

## Significance

Legged animals move from place to place without deviating from their desired movements despite inherent errors. Humans and some robots achieve such stability by placing the swinging foot in a manner that corrects errors. However, we do not understand how such stability is achieved by animals with diverse brains and bodies. Here, we find that other legged animals also move their swinging leg in response to inherent errors. We further find that the inherent mechanical stability of the animal’s body shapes aspects of its control strategy. Our approach to investigate strategies for stable locomotion across species can help advance comparative neuroscience and legged robotics.

Stable locomotion is a fundamental skill for legged species as it enables navigating the environment without time-consuming course corrections in the presence of intrinsic sensorimotor noise or external perturbations. However, we lack a unified understanding of how this important function is achieved in species with diverse neural control and body mechanics, i.e. across neuromechanical embodiment. In humans and legged robots, foot placement control is an important strategy for stable locomotion (1, 2), whereby the swinging leg is placed in a manner that corrects recent errors in the body state (3–5). However, we lack insight into analogous error-dependent foot placement control in multilegged animals, where foot placements are typically thought to be velocity-dependent (6, 7). Here, we hypothesize that a unified control structure underlies stable locomotion across species, combining velocity- and error-dependent contributions to control. By mining natural locomotor variability, we find that flies (Drosophila melanogaster), mice, and humans exhibit shared signatures of velocity- and body state error-dependent foot placement control. These findings enable comparative insight into the neuromechanics and motor control of stable locomotion.

Understanding the neuromechanical basis of motor control requires comparative insight into how different species achieve the same behaviorally relevant functional goal (8, 9). While the behavioral signatures of stereotypical velocity-dependent locomotor patterns across many species including humans (10–16) have helped solidify the existence of velocity-driven neural control during locomotion (6, 17), we lack analogous cross-species signatures of error-dependent locomotor control. Indeed, while there is agreement about the existence of error-dependent locomotor control in humans (3, 4, 18), we lack comparable evidence in other legged animals. Such control could rely on diverse sensory feedback (19–24) and neural processing (21, 25–27), but its mechanistic underpinnings are challenging to establish directly in humans. Thus, identifying common signatures of error-dependent locomotor control across species is an important step toward understanding its neuromechanical basis. To address this, we develop a unified data-driven model to identify shared foot placement control signatures across species. Using this model, we find that mice and flies exhibit human-like signatures of both error-dependent and velocity-dependent foot placement control.

Explaining how animals achieve stable locomotion has been a long-standing goal for theoretical modeling. Two primary theoretical approaches focusing on “feedforward” and “feedback” control, as defined in ref. 28, have emerged to explain locomotor stability. The first approach focusing on feedforward control posits that stable locomotion emerges from leg actuation based on a sparse top–down signal such as locomotor speed without the need for frequent sensory feedback, leveraging the intrinsic mechanical properties of the body to guarantee stability (29–32). In such theoretical and robotic models, passive biomechanical stability in combination with a controller that prescribes a low dimensional set of actions such as leg length, leg angle, or leg stiffness has been shown to be sufficient to guarantee stability (33–38). A second theoretical approach focused on feedback control suggests that feedforward strategies might be insufficient to guarantee stability during locomotion (39, 40), particularly in the lateral direction. These theoretical models incorporate recent feedback about the body state to take corrective actions (3, 40–42). Yet, such feedforward and feedback control has not, to the best of our knowledge, been unified into a single control structure to explain locomotion across species. Moreover, although both feedforward and feedback control structures have been shown theoretically to enable stable locomotion, there is insufficient empirical evidence to weigh in on this debate. To address this gap, we posit a unified feedforward-feedback control structure combining velocity-dependent and body state error-dependent foot placement control and test the ability of this control structure to explain natural locomotor variability in flies, mice, and humans.

An animal’s locomotion is shaped by its neuromechanical embodiment, namely the interplay between the mechanical properties of its body and its neural control strategies (28, 43, 44). Biomechanical factors like passive muscle mechanics (45), body size-dependent transmission delays (46, 47), and leg configuration (48, 49) can influence the chosen control strategies. Similarly, neural control during locomotion is influenced by spinal circuits (6, 50) as well as by supraspinal neural centers (51–55). While the influence of these diverse neuromechanical factors on feedforward, velocity-dependent locomotion is relatively well-established (7, 43, 45, 56, 57), how they affect error-dependent control strategies like foot placement control remains poorly understood. Indeed, identifying common behavioral signatures of error-dependent locomotor control in species with diverse neural control and biomechanics is a first step toward understanding its neuromechanical basis. Here, we introduce a data-driven model that identifies error-dependent signatures of foot placement control across species and analyze how these signatures vary with multilegged neuromechanical embodiment.

In this study, we investigate stabilizing foot placement control during walking across species with diverse neuromechanical embodiment, identifying common signatures of a hypothesized feedforward-feedback control structure. Our approach and findings have implications for comparative neuromechanics, comparative neuroscience, and bioinspired legged robotics.

## Results

We found that a similar feedforward-feedback structure underlies stabilizing foot placement control in any legged animal and identified signatures of this control structure hidden in natural locomotor variability. We characterized these signatures in flies (D. melanogaster), mice (C57BL/6J) and humans using high-throughput locomotion data, identifying shared velocity-dependent and body state error-dependent signatures across species, and highlighting how the signatures change with more inherently stable and multilegged embodiment.

### Feedforward-Only and Feedforward-Feedback Control Structure Hypotheses for Foot Placement.

We evaluate two hypothesized control structures underlying foot placement across species: a feedforward-only and a feedforward-feedback control structure. We use the terms “feedforward” and “feedback” here as defined in a recent review (28). Note that we use these terms to refer to the hypothesized input–output control structure without making more specific claims about the neural basis of the control. Also, we use the term foot to refer to the limb extremity, independent of differences in the limb anatomy across species, similar to how the term foot placement control is used in robotics (58, 59).

**Fig. 1.**
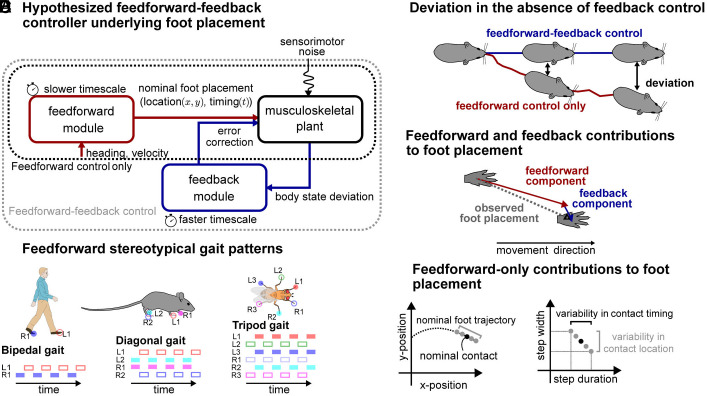
Hypothesized control structure for stable locomotion in legged animals. (*A*) Schematic representation of the two alternative control structures underlying foot placement as well as the interactions between the velocity-dependent feedforward control module (red) and the body state error-dependent feedback control module (blue). The feedforward module transforms velocity and heading inputs into nominal foot placements over multiple gait cycles. The feedback module corrects body state errors by making appropriate adjustments to the next foot placement. (*B*) Canonical gait patterns for humans, mice, and flies. Colored rectangles indicate the times at which each limb is in stance; full and empty rectangles illustrate the two groups of legs alternatively in contact with the floor during the specific gait patterns studied. (*C*) Hypothesized deviations in the body states due to intrinsic sensorimotor noise in the presence (blue) and absence (red) of error-based feedback control. (*D*) Under the feedforward-feedback assumption, the observed foot placement is the resultant of the individual contributions of feedforward and feedback modules. (*E*) The velocity-dependent nominal trajectory of the foot (red line, *Left*) prescribes the position of the foot as a function of time. A slightly shorter or longer contact timing could result in deviations of the contact location from its nominal position (gray dots versus black dot). Therefore, the variability in contact timing could result in apparent variability in contact location even in absence of body state error-based information.

The first control structure hypothesis we test is feedforward-only (Fig. 1*A*, black box), transforming velocity and heading inputs into nominal foot placements and body kinematics for each species, reflecting their characteristic gait patterns (Fig. 1*B*). We rationalize these inputs to the feedforward controller with animal neuroscience research, which suggests that velocity and heading are important descending inputs for the generation of periodic interlimb coordination patterns (60–62). We select the output actions for this feedforward-only controller as the foot placement location and timing, rationalized by prior work (37, 63). In doing so, we extend prior work, which focuses on feedforward control in the fore–aft direction, by testing whether velocity-dependent changes in the lateral foot placement location and timing could also have a feedforward stabilizing effect (Fig. 1*E*).

The second hypothesized control structure is hierarchical and feedforward-feedback, consisting of two different control modules (Fig. 1*A*, gray box): a velocity-dependent module and a body state error-dependent module. The body state error-dependent feedback module operates in the vicinity of the velocity-dependent feedforward module, to correct deviations from the velocity-dependent nominal body states including positions and velocities (Fig. 1*C*). This feedforward-feedback controller is hierarchical as each module has access to distinct inputs and operates at different timescales. The feedforward module transforms the velocity and heading inputs into nominal foot placement patterns over a timescale of multiple gait cycles, while the feedback control module transforms the body state errors into corrective foot placements at a faster timescale (Fig. 1*A*). Importantly, these body state errors are defined as deviations from the velocity-dependent nominal states and not from the average behavior as done in previous work (3, 64, 65), which allows us to unify error-dependent and velocity-dependent foot placement control. We rationalize the body state error-dependent feedback module with human locomotor stability research on error-based foot placement control in healthy (3, 18) and disordered locomotion (26, 27, 66–68). Per such a feedforward-feedback control structure, the observed foot placement would be the net resultant of the individual contributions of each control module (Fig. 1*D*).

If the feedforward-only controller is sufficient to explain foot placement, we should find that the velocity-dependent module explains an equal or greater amount of the variability in the foot placement location and timing than the body state error-dependent module (Fig. 1*E*). Alternatively, if the feedforward-feedback control structure underlies foot placement, we should find that the body state error-dependent module explains a significant amount of the variability in foot placement location and timing that is not captured by the velocity-dependent module alone (Fig. 1*D*).

### Signatures of Velocity-Dependent Feedforward Foot Placement Across Species.

The feedforward control module (Fig. 1*A*, red) posits that movement velocity contributes to the foot placement patterns adopted by animals. Although the specific contribution of velocity to both fore–aft and lateral foot placement variability has been characterized in humans (69), this relationship is less understood particularly for lateral foot placement across species. To simultaneously test our hypothesized feedforward module and understand the nature of its velocity dependence in multilegged animals, we took a data-driven approach, correlating the movement velocity and the foot placement location and timing variability.

**Fig. 2.**
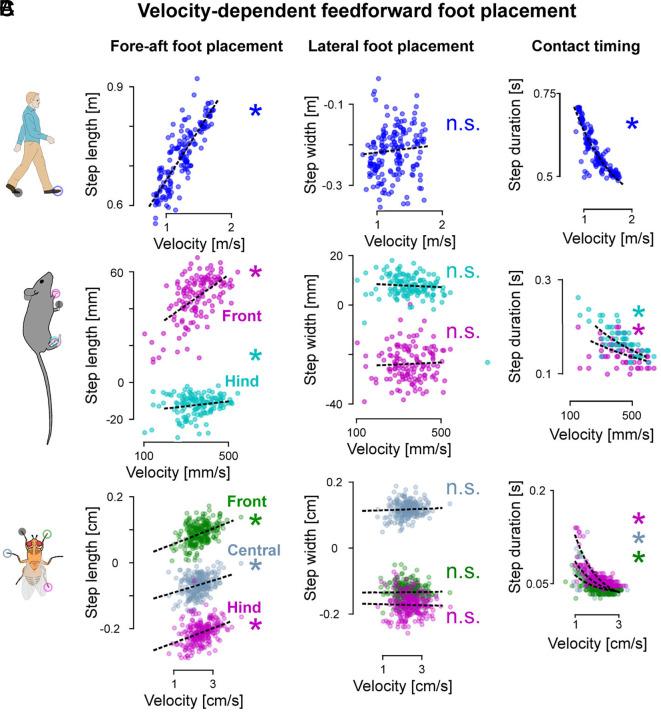
Signatures of velocity-dependent feedforward foot placement control across species. (*A*) Modulation of step length (*Left*), step width (*Center*), and step duration (*Right*) with movement velocity for humans. (*B*) Modulation of step length (*Left*), step width (*Center*), and step duration (*Right*) with movement velocity for front (magenta) and hind (cyan) limbs of mice. (*C*) Modulation of step length (*Left*), step width (*Center*), and step duration (*Right*) with movement velocity for front (green), central (blue), and hind (magenta) limbs of flies. The black dashed lines capture the individual regressions between movement velocity and the step width/length. The reference limbs are represented as black disk overlaid on the animal cartoon, the open disks represent the different feet studied. ∗:P<0.05.

We found a consistent positive correlation between movement velocity and step length across all species and limbs, but not between velocity and step width. In flies, step length for each leg is positively correlated with movement velocity (chatterjee-xi test: P<0.05 for all legs; Fig. 2*C*, *Left*) while step width shows no correlation (chatterjee-xi test: P=0.924 front, P=0.3445 middle, and P=0.138 hind; Fig. 2*C*, *Center*). Post hoc regressions confirmed that flies linearly increase their step length with increasing movement velocity (significant in 8/8 videos for front, middle, and hind limbs). Mice also show a positive correlation between step length and velocity (chatterjee-xi test: P<0.05 for all legs; Fig. 2*B*, *Left*), but not step width (chatterjee-xi test: P=0.15 front and P=0.0636 hind, Fig. 2*B*, *Center*). Similar to flies, mice linearly increase their step length with increasing velocity for both front (significant in 79/80 animals) and hind limbs (significant for 44/80 animals). In humans, we observed a similar pattern: Step length increases with increasing velocity (chatterjee-xi test: P<0.05, post hoc significant for 20/21 subjects, Fig. 2*A*, *Left*), while step width remains constant (chatterjee-xi test: P=0.06, Fig. 2*A*, *Center*). Finally, we found a negative correlation between step duration and velocity (chatterjee-xi tests: P<0.05 for all legs and all species, Fig. 2*A*–*C*, *Right*) and found that step duration exponentially decreases with increasing velocity across all species.

The step width variability that is not explained by movement velocity could stem from variability in contact timing, as suggested by the feedforward-only controller (Fig. 1*E*). To investigate this possibility, we tested for potential correlations between the deviations in contact timing from its nominal behavior (Fig. 2, *Right* column) and the deviations of the step width from its nominal behavior (Fig. 2, *Central* column). We did not find such correlations for any of the species (chatterjee-xi test: P>0.05), which suggests that the feedforward-only hypothesis is not the sole source of the observed step width variability.

Thus, we found a similar influence of movement velocity on nominal foot placement across all species and limbs. Specifically, movement velocity modulates both step length and step duration but not the step width in all species. Foot placement timing variations also do not explain the variance in step width. Faster movement velocities result in longer steps and shorter step durations, while step width remains largely unaffected by the hypothesized feedforward-only control structure.

### Signatures of Body State Error-Dependent Feedback Foot Placement Across Species.

**Fig. 3.**
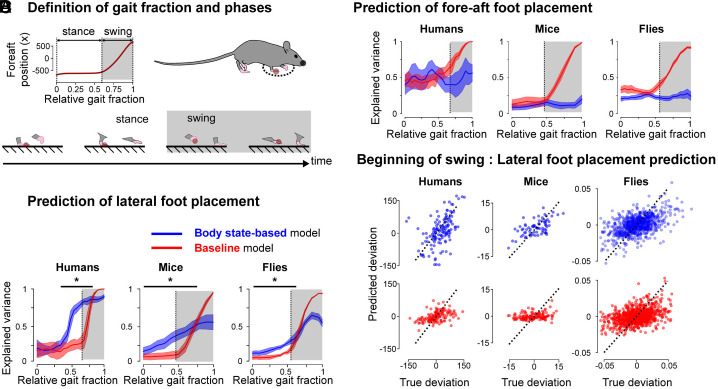
Signatures of body state error-dependent feedback foot placement control. (*A*) We defined the gait fraction as the time elapsed since the last contact of the reference foot divided by the duration of the gait cycle. The stance and swing phase of each foot (here illustrated for the foot highlighted in red) were defined as the periods during which the foot was or was not in contact with the ground, illustrated here for one of the front limbs in mice. The swing–stance and stance–swing transitions correspond to contact initiation and contact termination. We built models to predict future contact initiation based on the kinematics of the body during the gait cycle preceding that contact. (*B*) Explained variance (R^2^, median, and interquartile range) obtained for the body state-based and baseline predictions of the lateral deviation in foot placement as a function of the relative gait fraction. The gray areas represent the swing phase of the foot to be placed, the vertical dashed lines represent the stance–swing transition for each species. (*C*) Explained variance (R^2^, median, and interquartile range) obtained for the body state-based and baseline prediction predictions of the fore–aft foot placement as a function of the relative gait fraction. The gray areas represent the swing phase of the foot to be placed, the vertical dashed lines represent the stance–swing transition for each species. (*D*) Comparison between the true and predicted deviations for the body state-based (*Top*, blue) and baseline (*Bottom*, red) predictions of the lateral foot placement of humans, mice, and flies front limbs at the beginning of the swing phase of the contact foot [dashed line in panel (*B*)]. The black dashed lines represent the identity lines corresponding to perfect prediction. ∗:P<0.05.

Our hypothesized feedback module (Fig. 1*A*, blue) suggests that errors in the body state on each step should inform the future foot placement. To test this hypothesis and characterize the feedback control module, we mapped the kinematic body state errors (i.e., positions and velocities) with the future foot placement, quantifying the variance in foot placement explained by this mapping as a function of gait fraction. Such a mapping has previously been identified during human treadmill locomotion (3, 5, 70), but its existence in other legged species and during natural overground human locomotion remains unknown. To characterize this mapping across legged species, we calculated body state and foot placement errors, defined as deviations from the nominal patterns predicted by the velocity-dependent model, at various gait fractions (Fig. 3*A*) and identified linear relationships between the deviations in the body state and the foot placements (*Materials and Methods*). We then compared these mappings to a baseline mapping derived from foot kinematics alone to determine whether body state errors contain more information about future foot placement than the foot kinematics itself.

We found that body state errors predict future lateral foot placement in the front limbs of flies, mice, and humans. In all species, these body state error-based predictions outperform the baseline predictions, derived from the foot kinematics, well before swing onset. In flies, the body state-based predictions were superior (Kolmogorov–Smirnov test P<0.05, Fig. 3*B*, *Right*) from 0% to 65% of the gait cycle, while swing onset occurs around 55%. In mice, the body state error-based predictions were superior (Kolmogorov–Smirnov test P<0.05, Fig. 3*B*, *Center*) from 0% to 70% of the gait cycle, while swing onset occurs around 50%. In humans, the body state error-based predictions were superior (Kolmogorov–Smirnov test P<0.05, Fig. 3*B*, *Left*) from 30% to 80% of the gait cycle, while swing onset occurs around 65%. In all three species, the body state-based model predicts deviations more accurately than the foot kinematics–based model well before swing onset (Fig. 3*C*, representative data). We also analyzed the fore–aft foot placement in a similar manner and found no significant differences across all three species (Fig. 3*C*); the body state error-based model prediction is never better than the baseline model for fore–aft foot placement.

We also analyzed the eigenvalues of the Poincaré return maps for all three species. Eigenvalues with a modulus lower than 1 indicate a stable gait. We find that the modulus of all eigenvalues is indeed smaller than 1 for all three species, with the spectral radii (the modulus of the largest eigenvalue) decreasing with increasing number of legs (humans ρ=0.98, mice ρ=0.65, and flies ρ=0.48). To determine whether the body state error-based foot adjustments contribute to stability, we next investigated whether lateral variations in foot placement contribute to error reduction. We reason that if these variations play a stabilizing role, larger foot placement deviations should be associated with larger reductions in body state errors from one half-gait cycle to the next (*Materials and Methods*). We found a significant positive correlation between foot placement deviation and subsequent error reduction in all three species (humans: P<0.05 and β=0.49, mice: P<0.05 and β=0.29, flies: P<0.05 and β=0.87, *SI Appendix*, Fig. S5). Notably, this analysis between foot placement deviation and error reduction can be reformulated as differences-in-differences, which is a quasi-experimental causal inference approach (*SI Appendix*, section S3 for details). This finding suggests that the observed mappings between body state error and lateral foot placement can have a stabilizing effect on the body.

Thus, we found that body state errors predict future lateral, but not fore–aft, foot placement in the front limbs of mice and flies, similar to humans. We also provide evidence that these error-based foot placements could have a stabilizing effect, reflected in the finding that past foot placement magnitude is correlated with future reduction in body state errors. These findings are consistent with the body state error-based control structure hypothesis for lateral foot placement during natural locomotion across species.

### Signatures of Control Magnitude and Timescale Vary Across Embodiment.

**Fig. 4.**
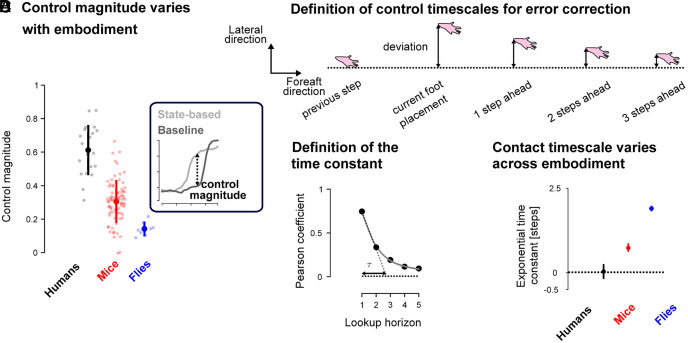
Magnitude and timescale of the control signatures vary across embodiments. (*A*) Group median and interquartile range of the control magnitude for the lateral foot placements of humans legs (black), mice front legs (red), and flies front legs (blue) at the initiation of the swing phase of the contact foot. The control magnitude is defined as the difference between the amount of variance explained by the body state-based and the baseline prediction (*Inset*). (*B*) Schematic representation of the variations in the lateral foot placement as a function of time. We computed the correlation between the current lateral foot placement and the subsequent lateral foot placements. The Pearson coefficient of correlations exponentially decayed with the look up horizon as schematized in panel (*C*). We characterized this exponential decay by the exponential time constant, τ. (*D*) Exponential time constants in humans, mice, and flies (maximum across legs for each species). The error bar represents the centered 90% CI based on bootstrap resampling.

We have found qualitatively similar body state error-dependent lateral foot placement control signatures, hidden in locomotor variability, in flies, mice, and humans. However, the details of the control strategy—such as how much error is corrected and over what timescale (71)—may vary across species due to more passively stable embodiment afforded by multiple limbs. To understand how the amount of error corrected in each step, which we termed the control magnitude, varies with embodiment we computed the maximal difference between the coefficient of determination of the body state error-based model relative to the foot trajectory-based baseline model (Fig. 4*A*, *Inset*) for each individual regression, and compared it across species. We observed a gradient across species (Fig. 4*A*) with the largest differences observed in humans (0.61 ± 0.14), followed by mice (0.31 ± 0.12) and by flies (0.14 ± 0.04). These results suggest that, although these species leverage qualitatively similar body state error-dependent foot placement signatures, the extent to which errors are corrected in a single step varies with many-legged embodiment. Specifically, the explained variance by body state error-based control decreases with increasing passively stable embodiment.

While we found that the body state error-dependent foot placement control magnitude was lower for more passively stable animals, this did not tell us about the control timescale, i.e., over how many foot contacts the error was corrected. To capture that control timescale, we computed the correlations between a given lateral foot placement of a limb and its subsequent lateral foot placements (Fig. 4*B*). These correlations, captured by the Pearson coefficient, exponentially decayed with time (Fig. 4*C*, illustrative data). We extracted the maximal exponential time constant (τ in Fig. 4*C*), across limbs, for each species and observed a gradient across them (Fig. 4*D*) with the smallest time constants observed in humans (0.035 ± 0.43), followed by mice (0.73 ± 0.2) and flies (1.89 ± 0.11). These results suggest that the timescale over which errors are corrected varies with many-legged embodiment—the correction timescale increased with the number of legs.

Thus, we found that the magnitude and timescale of the lateral foot placement control signatures vary with many-legged embodiment; with a larger number of limbs, the magnitude decreases, and the timescale becomes increasingly multistep.

### Signatures of Direction- and Leg-Specific Modular Control of Foot Placement.

We have found shared signatures of body state error-dependent foot placement control from locomotor variability in many-legged animals, and quantified how the control magnitude and timescale vary with embodiment. With the availability of multiple legs to execute the same functional goal, namely stable locomotion in the presence of sensorimotor noise, animals may use a more or less centralized control strategy, i.e., they may use different legs or muscle groups for executing modular control functions (72). Here, we analyzed the locomotor variability to test the existence of such leg-specific or direction-specific modular control.

**Fig. 5.**
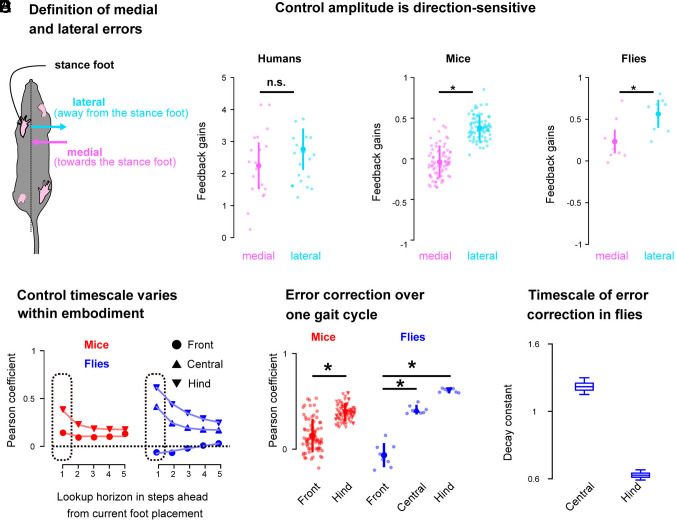
Foot placement control signatures reveal direction-specific and limb-specific modularity across species. (*A*) We defined lateral (cyan) and medial (magenta) errors respectively as the errors directed away and toward the stance foot. We independently fitted models for lateral and medial errors and compared their foot placement control gains. (*B*) Group median and interquartile range of the lateral (cyan) and medial (magenta) foot placement control gains for humans, mice, and flies at the beginning of the swing phase of the contact foot. (*C*) Pearson correlation coefficients as a function of lookup horizon for the different limbs in mice (red) and flies (blue), group median. (*D*) Group median and interquartile range of the initial Pearson correlation coefficient [see dashed rectangle in panel (*C*)] for the different pairs of limbs in mice and flies. (*E*) Timescale of the error corrections for central and hind limb in flies, the error bars capture the 90% CI. ∗:P<0.05.

We hypothesized that, with the availability of multiple legs for control, medial, and lateral body state errors relative to the stance foot, i.e., errors that are directed toward and away from the stance foot respectively, would be controlled differently (Fig. 5*A*). The rationale for this hypothesis is that medial errors in the body state near the frontmost limbs directed toward the stance foot can be corrected by the impedance of the stance foot itself, unlike lateral errors directed away from the stance foot and toward the swinging foot. To test this hypothesis, we computed the feedback gains associated with medial and lateral body errors separately (*Materials and Methods*) and compared them at the beginning of the swing phase of the foot being placed. Medial and lateral feedback gains were similar in humans (Fig. 5*B*, *Left*, Wilcoxon signed-rank, P=0.13, d=0.38) but differed in mice (Fig. 5*B*, *Center*, Wilcoxon signed-rank, P<0.05, d=1.57) and flies (Fig. 5*B*, *Right*, Wilcoxon signed-rank, P<0.05, d=1.20), where the corrections were larger for lateral than for medial errors.

Within a given embodiment, we posit that different pairs of limbs could have different control strategies; the rationale for this hypothesis is that in quadrupedal animals, the front and hind limbs are innervated by different spinal circuits and therefore may exhibit distinct behavioral control signatures (17). To test the existence of such leg-specific differences in control strategies, we compared the mapping between the body state errors and lateral foot placement across different limb pairs—from anterior to posterior as defined by the movement direction—over multiple gait cycles (Fig. 5*C*). We observed that the correlations over a single gait cycle (see dashed *Insets* in Fig. 5*C*) varied across legs; larger values were observed for more posterior limbs in both mice (Fig. 5*D* red, Wilcoxon signed-rank test, P<0.05, d=1.37) and flies (Fig. 5*D* blue, Kruskal–Wallis with post hoc Dunn’s test and false detection rate corrections, front-central : P<0.05, d=1.88, front-hind : P<0.05, d=1.94). The exponential time constant associated with the exponential decay of these correlations also varied across limbs in flies where the hind limbs had a smaller constant, i.e., a slower correction timescale, than the central limbs (Fig. 5*E*). These results suggest that the control strategies for lateral foot placement in different limbs of flies and mice operate with different timescales—the more posterior pairs of limbs operate at slower timescales and have persistent control across multiple contacts.

Thus, we found that the control strategies used for foot placement by many-legged species reveal direction- and leg-specific modular control, exhibiting variation with error direction along the medial–lateral axis, and with limb pairs along the anterior–posterior axis relative to movement direction. We found that both the control gains and timescales exhibit such leg- and direction-specific modularity.

## Discussion

We put forth a unified feedforward-feedback control structure underlying foot placement for stable locomotion in any legged animal. Based on this control structure, we develop a data-driven model to identify signatures of control from natural locomotor variability across species. Using this model, we find signatures of body state error-dependent locomotor control in flies and mice, previously only shown to exist in humans. We find that the urgency of foot placement control, reflected by the control magnitude and timescale, decreases in more passively stable embodiment. Foot placement control is executed modularly in many-legged animals as illustrated by the use of limb- and direction-specific gains and timescales. These findings have implications for understanding the neuromechanics of animal locomotion, uncovering the neural basis of stabilizing control, developing cross-species phenotypes for locomotor control, and bioinspired legged robots.

### Foot Placement Control Signatures in Flies, Mice, and Humans.

We found signatures of body state error-dependent control from unperturbed locomotor variability in multilegged animals, a phenomenon previously only observed in humans (3, 4, 70). We put forth a shared control structure for foot placement across species, unifying feedforward and feedback contributions to control. Our feedforward control module captures the stereotypical gait patterns of different species and how they covary with movement velocity. Our feedback control module, which captures the body state error-dependent corrective foot placements, echoes previous work highlighting the importance of lateral foot placement for stable locomotion in bipeds (3, 18), and provides cross-species body state error-dependent signatures of foot placement control, previously posited in theoretical models of legged locomotion (40, 73). Moreover, while previous work reported similarities in the structure of kinematic variability between mice and flies (74, 75), our work demonstrates how this variability relates to stability by correlating foot placement to recent body state errors. Here, by eliminating the velocity-dependent component of foot placement, our data-driven model is able to avoid erroneous interpretation of body state error-dependent foot placement in the fore–aft direction (Fig. 3*C*), supported by theoretical modeling (65). Overall, our findings are consistent with body state error-dependent lateral foot placement control across species.

### Empirical Support for the Control Structure Underlying Foot Placement.

Using a data-driven model inferred from natural locomotor variability, we evaluated two competing hypothesized control structures for legged locomotion. The first structure posits purely velocity-dependent feedforward control (Fig. 1*A*, red), while the second (ours) also posits body state error-dependent feedback control in the vicinity of the feedforward controller (Fig. 1*A*, red+blue), contrasting previous data-driven studies that defined such feedback control in the vicinity of the average behavior (3, 64, 65). Our data-driven signatures in humans, mice, and flies are consistent with the feedforward-feedback control structure. Although both theorized control structures explain the data-driven velocity-dependent component of foot placement location and timing, only the feedforward-feedback model can explain the lateral foot placement variability (Fig. 3*B*). Thus, we provide cross-species empirical support consistent with the hypothesis that velocity-dependent control is insufficient for lateral stability (40, 73). In this work, we more clearly link the body state error-dependent feedback control signatures to their stabilizing effect on the body by identifying a positive correlation between corrective foot placements on one step and subsequent error reduction for the body state on the next step. While our data-driven behavioral signatures alone do not guarantee the existence of causal body state error-dependent control during locomotion, when viewed in combination with previous theoretical work (18, 39–41, 73), it puts forth a more compelling case by providing missing empirical support. Future work could use our framework to further characterize the inputs and outputs of each control module by determining for instance whether the speed, the relative gait timing, or both are the more relevant inputs to the feedforward control module or by identifying whether the feedforward module directly controls the foot contact or the swing leg kinematics (37, 38).

### Neuromechanical Basis of Foot Placement Control.

The signatures of foot placement control we found could help investigate the neuromechanical basis of stable locomotion across species. The hypothesized feedforward-feedback control structure suggests distinct yet interacting mechanisms for velocity-dependent and body state error-dependent control. The signatures of velocity-dependent fore–aft foot placement and contact timing (Fig. 2) aligns with the periodic velocity-dependent coordination afforded by pattern-generating neural circuits (6, 17, 50). The lateral body state error-dependent foot placement (Fig. 3) suggests an additional feedback mechanism, which we speculate could map to multisensory feedback-guided steering at different hierarchical levels (see *SI Appendix*, section S4 for an elaboration). We posit that such feedforward and feedback control mechanisms could interact because our model’s feedback module relies on information about the nominal body state from the feedforward module (Fig. 3*B*). We speculate that such body state error estimation in mice and humans could rely on vestibular information (53–55) mediated through supraspinal neural centers (76–82), whose disruption affects locomotor control (52, 83). The observed cross-species differences in foot placement control amplitudes and timescales (Fig. 4) suggest links to the differences in neuromechanical embodiment. While our data-driven models alone cannot definitively distinguish between the contributions of biomechanics, neural circuitry, or both, to the observed differences in foot placement control across species, these findings open exciting avenues for future research. For example, combining our approach with mechanical (49, 84) or neural perturbations (54) could help isolate the neural and biomechanical contributions to foot placement control. Alternatively, our data-driven model could be combined with physics-based imitation learning to posit the neuromechanical basis of the observed signatures in simulation (85, 86). These findings can also inform the development of cross-embodiment control strategies in legged robots (87), by providing bioinspiration for the selection of neuromechanical control primitives (Fig. 5). Our data-driven model could also be used in combination with recent simulation work to identify which sensory inputs are associated with the feedforward- and feedback-specific control signatures we find (85, 88, 89). Such work could determine whether, for instance, vision is primarily used by the feedforward module (90), or vestibular information is primarily used by the feedback module (54). Overall, understanding how foot placement control strategies change with neuromechanical embodiment across species can provide insight into the coevolution of biomechanics and neural control.

### Inferring Control from Intrinsic versus External Perturbations.

Here, we investigate the control strategies hidden in natural locomotor variability that ensure stability in the presence of intrinsic sensorimotor noise-like perturbations as opposed to externally applied perturbations. Such a “default controller” (91) to handle perturbations originating from intrinsic errors is ethological, because all animals likely have a degree of unavoidable sensorimotor noise that continuously perturbs their movements (92, 93), which they must remain stable to. Indeed, in humans, studying this default controller hidden in natural variability has been important for understanding foot placement control (3, 5, 91), revealing that this controller is robust enough to handle larger perturbations (5, 64). Inferring control from natural variability also offers a link to numerous mathematical formulations of stability (94), following the example of how these formulations have been linked to variability in human locomotion (4). For instance, the signatures of body state error-dependent feedback control in multilegged animals, as found here, are reminiscent of the center of mass-based control in humans and suggests that stability formulations developed for abstract inverted pendulum models (18) or more detailed robotic models (95) could be applied across species. Furthermore, our finding of slower control timescales in more inherently stable animals align with previous work on the impact of leg number and configuration on dynamic stability (73). Finally, our findings of embodiment-dependent control timescales can be linked to theoretical studies that posit the number of steps required to reach a stable body state (71, 96–98) which is related to falls in humans (99). By deriving control signatures from natural variability, we can bridge the gap between theoretical models and natural locomotion across species.

### Weaknesses and Strengths of Studying Control from Natural Variability.

Our work provides a way to compare control strategies across species for a shared ethological goal: foot placement control in the presence of intrinsic sensorimotor noise. Investigating animal locomotion in such an ethological setting has both limitations and strengths. One key limitation is the inability to know, from behavior alone, the precise nature of the noise-like input which results in errors to be controlled. Here, we hypothesized that the observed foot placement variability is the result of feedforward-feedback control in response to intrinsic sensorimotor noise (Fig. 1*A*), which has some theoretical basis (5, 93), but confirming whether this noise is motor, sensory, or neural in origin is challenging with theory and behavior alone (100). Similarly we hypothesized that the differences we observed across species (Fig. 4) are related to the differences in their neuromechanical embodiment, but our natural variability-based data-driven model cannot discriminate between biomechanical and neural contributions to these differences. These challenges are an opportunity for future neuromechanics research to uncover the source of intrinsic sensorimotor errors and that of the cross-species differences in foot placement control. Even if we only analyzed walking gaits, i.e., gaits for which there is no flight phase, we suspect that our approach could generalize to faster gaits in mice and flies, as it did in humans (5). However, the reliance on body state error-based control could significantly decrease at high speeds (101–103) because of the lack of time to process delayed sensory inputs. There are also strengths to studying the control strategies underlying natural variability, such as the discovery of ethological control principles, the relevance to easily quantifiable clinical biomarkers, and the ease of reproducibility. Since all animals have some degree of intrinsic sensorimotor noise, inferring control strategies from natural variability is ethological and representative of the animal’s natural control repertoire (104, 105). Population differences in human locomotor variability are used as biomarkers for neuromotor disorders that impair balance, such as Parkinson’s disease (66). Here, we have shown that neuroscientifically accessible animal models like flies and mice exhibit human-like signatures of control hidden in variability, thus encouraging the study of these control signatures as potential cross-species biomarkers. Our approach is easily reproducible as it is applicable to any multilegged animal (*Materials and Methods*), builds on open source video processing pipelines (106, 107), and does not require specialized equipment other than a camera placed such that foot contacts are discernible. Moreover, as locomotion is a behavior that is readily exhibited by animals, our approach can enable post hoc analyses of large-scale animal behavior datasets (52).

### Conclusion.

This work establishes three key findings: i) cross-species signatures of body state error-dependent foot placement control across species, ii) how these control signatures change with neuromechanical embodiment, and iii) data-driven signatures consistent with a combination of velocity-dependent and body state error-dependent control, and inconsistent with purely velocity-dependent control in flies, mice, and humans. These findings provide a starting point for future investigations into the neuromechanical basis of foot placement control across species.

## Materials and Methods

### Mathematical Formulation of Feedforward-Feedback Foot Placement Control.

We hypothesize that foot placement control in many-legged animals is generated by a feedforward-feedback control structure (Fig. 1*A*) composed of a velocity-dependent feedforward module and a body state error-dependent feedback module. This control structure outputs the net observed foot placement location and timing as the resultant of the contribution of its two control modules (Fig. 1*D*): The feedforward module contributes the foot placement prescribed by the canonical gait patterns (74) while the feedback module contributes body state error-based corrective adjustments of foot placement (3). These modules operate at different timescales and are organized in a *hierarchical* manner such that each of them has access to some privileged information which is not available to the other. The input to the feedforward control module is the average velocity during the last gait cycle $\hat{v}$ while the inputs to the feedback control module are the body state deviations from the stereotypical species-specific gait pattern $Q^{*}$, expressed as phase-dependent body state errors $\Delta Q\left(\phi \right)=Q\left(\phi \right)-Q^{*}\left(\phi \right)$, where ϕ is the relative gait fraction defined as the ratio between the duration since the beginning of the last gait cycle and the total duration of that gait cycle. For each step, the net observed foot placement is expressed as the sum of the individual contributions from the two control modules

[1] $$\begin{array}{c} P=f\left(\hat{v}\right)+g\left(\Delta Q\left(\phi \right)\right), \end{array}$$

where functions f and g are respectively the contributions of the feedforward and feedback control modules. We used the data from naturalistic locomotion of different legged species to investigate signatures of the hypothesized control structure across species.

### Inference of Control Signatures from Locomotion Data.

We combined the hypothesized control structure underlying legged locomotion with a data-driven model to investigate signatures of feedforward and feedback foot placement control. First, we investigated the velocity-dependent component of foot placement in the fore–aft and lateral directions. To do this, we generalized the methodology used in humans to many-legged embodiment (69): For each gait cycle, we defined the movement velocity as the average fore–aft velocity of the body during the last gait cycle. We observed that the relationships between the movement velocity and the fore–aft and lateral foot placement were linear (Fig. 2*A*–*C*) and characterized this relationship with the model

[2] $$\begin{array}{c} f_{j}^{x,y}\left(\hat{v}\right)=\beta_{0,j}^{x,y}+\beta_{1,j}^{x,y}\hat{v}, \end{array}$$

where $\beta_{0,j}^{x,y}\;\text{and}\beta_{1,j}^{x,y}$ are scalar model parameters capturing the relationships for the jth leg along the fore–aft (x) and lateral (y) direction. We used individual models for each animal/subject and for each limb to capture how that limb’s step length and width were modulated by the average velocity. We observed that the relationships between movement velocity and the contact timing followed an exponential function (Fig. 2*E*–*G*) and characterized this relationship with the model

[3] $$\begin{array}{c} f_{j}^{t}\left(\hat{v}\right)=\beta_{0,j}^{t}\exp\left(-\beta_{1,j}^{t}\left(\hat{v}-\beta_{2,j}^{t}\right)\right)+\beta_{3,j}^{t}, \end{array}$$

where $\beta_{\dot{,}j}^{t}$ are scalar model parameters capturing the relationship for the jth leg.

To investigate the behavioral control signatures of the hypothesized feedback module, we defined the body state errors as the difference between the body state kinematics (positions and velocities) during the last gait cycle $Q\left(\phi \right)$ and the velocity-dependent body states as specified by the stereotypical gait pattern $\Delta Q\left(\phi \right)=Q\left(\phi \right)-Q_{\hat{v}}^{*}\left(\phi \right)$, where $\hat{v}$ is the average velocity over that gait cycle. We defined the corrective foot placements as the difference between the net observed foot placement P and the ones predicted by the feedforward control module $\Delta P=P-f^{x,y}\left(\hat{v}\right)$. We observed linear relationships between the body state errors and the corrective foot placements (Fig. 3*B*), which is theoretically valid for small errors, because continuous and differentiable nonlinear functions can be approximated as linear for infinitesimal changes. We investigated these relationships for the jth leg by the model

[4] $$\begin{array}{c} \Delta P\approx g_{j}^{x,y}\left(\Delta Q\left(\phi \right)\right)=\gamma_{0,j}^{x,y}+\sum_{i=1}^{N}\gamma_{i,j}^{x,y}\Delta Q\left(\phi \right), \end{array}$$

where $\gamma_{i,j}^{x,y}$ are scalar model parameters and N is the dimensionality of deviation vector.

We investigated the behavioral signatures of the feedback control module across species by comparing their amplitude and timescale. The control amplitudes were quantified as the maximal individual differences between the amount of variance explained by the body state error-dependent feedback model (blue traces in Fig. 3*B*) and that of a baseline model predicting the variability in foot placement from the foot kinematics itself (red traces in Fig. 3*B*). This baseline model, expressed as a function of the deviations in body kinematics $\Delta \tilde{Q}\left(\phi \right)=\tilde{Q}\left(\phi \right)-{\tilde{Q}}^{*}\left(\phi \right)$, was formalized as

[5] $$\begin{array}{c} \Delta P\approx {\tilde{g}}_{j}^{x,y}\left(\Delta \tilde{Q}\left(\phi \right)\right)={\tilde{\gamma }}_{0,j}^{x,y}+\sum_{i=1}^{N}{\tilde{\gamma }}_{i,j}^{x,y}\Delta \tilde{Q}\left(\phi \right). \end{array}$$

We then investigated the control timescales by correlating successive lateral positions of each foot over multiple gait cycles. The limb-specificity of the feedback control module was investigated by separating the medial and lateral errors and using the feedback model described above. Consistent with anatomical definitions, these direction-dependent errors $\Delta Q_{p_{y}}\left(\phi \right)$ are defined as follows: Medial errors are directed toward the body, while lateral are directed away from the body (Fig. 5*A*).

We assessed the locomotion stability using Poincaré return maps applied to the deviations in body kinematics. We defined these return maps based on the following relationship

[6] $$\begin{array}{c} Q\left(\phi =0.75\right)=KQ\left(\phi =1.75\right), \end{array}$$

where K is a scalar matrix evaluated based on the data. The eigenvalues of this matrix, $\lambda_{i}$, were computed for each species and the spectral radius defined as $\rho \left(K\right)=\;\max_{i}\left|\lambda_{i}\right|$ was reported to characterize the stability of the system.

We computed the variations in body state errors around contact as the difference between the averaged body errors during the half-gait cycle before and after contact based on the following relationship

[7] $$\begin{array}{c} \Delta \;\text{error}=\;\text{mean}\left(\Delta Q\left(\phi =0.5:1\right)\right)-\;\text{mean}\left(\Delta Q\left(\phi =1:1.5\right)\right) \end{array}$$

### Datasets Used to Investigate Foot Placement Control Across Species.

The contributions of the feedforward and feedback control modules can be inferred for any dataset for which kinematics data of the body and feet are available. To demonstrate this flexibility, we chose to use existing open source datasets of flies, mice, and humans during natural locomotion collected across different research groups.

The fly locomotion dataset was collected by DeAngelis and colleagues (75) with a Point Grey camera (Flea3-U3-13Y3M-C, 150 Hz) when 8 groups of 12 to 15 flies (*D. melanogaster*) were evolving in an open-field arena during 1.1 h. The data were segmented into individual locomotion bouts and the body and feet positions, as well as the individual foot contacts were extracted by the original authors using a custom-built markerless motion capture method (see ref. 75 for more details). We used the feet and the head markers in our analyses and only considered the segments during which the animals were not stopping. The mouse locomotion dataset was collected by Klibaite and colleagues (52) with a Point Grey grayscale camera (12-bit grayscale, 1,280 × 1,024 pixels, 80 Hz) when mice (C57BL/6J) were moving in an open-field arena on 4 consecutive days, 20 min each day. The positions of the different body parts were extracted using LEAP (107), an open source markerless pose estimation software. We used the feet, nose, and base of the tail markers for our analyses and only considered the data segments labeled as locomotion. The human locomotion dataset was collected by Camargo and colleagues (108) using 32 motion capture markers placed according to the Helen Hayes Hospital marker set (Vicon. Ltd., Oxford, UK). The data were collected at a frequency of 200 Hz and were filtered using a fourth order zero-lag Butterworth filter (cutoff frequency 6 Hz) during overground walking. We used the pelvis position, defined as the average position of the four hip markers, and the foot position, approximated by the position of the heel markers. We also used another human locomotion dataset collected by Wang and Srinivasan (3) for the analyses of the control timescales in humans (Fig. 4*D*) because the Camargo dataset lacked long locomotion bouts, required for these analyses. The data collection procedure was the same as that of the Camargo dataset and we used the same set of markers.

### Processing Pipeline for Obtaining the Inputs and Outputs of the Foot Placement Controller.

In this section, we describe how we processed the raw data described above to transform them into the inputs and outputs to the feedforward and feedback control modules. We sought to use an identical processing pipeline for all the datasets. In line with this, the steps outlined below were applied to all the datasets and species, unless otherwise stated.

We were interested in studying the straight-line locomotion bouts since foot placement control has primarily been characterized during straight-line locomotion in previous theoretical and human literature (2, 3, 18). We therefore selected the locomotion bouts for which the body orientation, defined as the direction of the instantaneous velocity vector in the horizontal plane, did not vary by more than 30°. We then rotated these straight-line locomotion bouts to align the heading direction across bouts, since the raw data contained locomotion bouts that can have different orientations in the world coordinates. We next identified the timing of each foot contact to extract the contact locations and segmented the gait cycles. For the datasets in which the contact timings were not provided, we computed the distance between the feet and body markers in the horizontal plane and defined the beginning and end of the stance phases as the maxima and minima of that distance (109). The predictions of contact locations obtained with this method were similar to those obtained with a velocity-thresholding of the foot marker (*SI Appendix*, Figs. S1 and S2), while being less sensitive to noise. We defined the contact locations, $P={\left[p_{x},p_{y}\right]}^{T}$ as the average positions of the foot markers during their stance phases and the contact timings as the time at which the swing to stance transition occurs. We used the contact initiations i.e. the beginning of the stance phases to perform the gait segmentation, defining a gait cycle as the interval between two successive contact initiations of the same limb. We performed this gait segmentation independently for each limb and grouped the gait cycles associated with the same limb. We compared the results obtained with a gait segmentation based on successive contact terminations of the same limb and did not observe any difference (*SI Appendix*, Fig. S3).

Because the inputs to the feedback control module are posited to be phase-dependent $\Delta Q\left(\phi \right)$, we temporally normalized the segmented gait cycles by linearly interpolating their marker positions and velocities onto a fixed set of discrete phase ϕ. The number of phases for each species was set to the average number of frames per gait cycle in the raw data. We validated this approach by comparing the predicted phase to the Phaser algorithm (110) which estimates the gait phase by combining the individual contributions of each leg; there was no systematic bias or difference between the predictions of these two approaches (*SI Appendix*, Fig. S4). Therefore, body and foot states were represented relative to phase ϕ, with ϕ=0 corresponding to the beginning of the gait cycle and ϕ=1 to the end of the gait cycle, which is when the contact corresponding to foot placement occurs. We then spatially normalized the marker positions and the contact locations by subtracting the last contact location of the front limb belonging to the opposite group of limbs. We defined the opposite group of limbs based on the canonical gait patterns whereby two groups of limbs are moving in opposite phase during walking (filled and empty rectangles in Fig. 1*B*).

### Statistical Analyses.

All the statistics were performed using Python3 (*numpy*, *scipy*, and *scikit_posthoc*). We used nonparametric tests whenever the normality condition, assessed with the Shapiro–Wilk test, was not met for at least one of the species. We used the Kruskal–Wallis followed by Dunn’s post hoc with FDR (false detection rate) corrections for multiple comparisons. We calculated and reported the effect size, quantified by the Cohen’s d for each comparison of two variables. We reported significance when the p value fell below 0.05.

We used the chatterjee-xi coefficient (111) to investigate correlations between pairs of variables. Chatterjee’s coefficient of correlation was chosen over Pearson’s coefficient as it is not restricted to monotonic trends. The chatterjee-xi test from *scipy* was used to test whether the variables were independent. We studied the specific impact of movement velocity on the step length and width (Fig. 2) by fitting individual linear regressions, motivated by previous studies (10, 69). We used the function *scipy.stats.linregress* in Python to fit these regressions and kept track of the individual slopes and p values associated with each of them.

To infer the feedback control module (Fig. 3), we obtained linear models between the body errors $\Delta Q\left(\phi \right)$ and the foot placement deviations ΔP. The best fit model parameters, obtained by the minimization of the mean squared errors, were given by $\hat{K}\left(t\right)=\frac{\Delta Q{\left(\phi \right)}^{T}\Delta P}{\Delta Q{\left(\phi \right)}^{T}\Delta Q\left(\phi \right)}$ and the coefficient of determinations were given by $R^{2}=1-\frac{\Sigma r^{2}}{\Sigma \Delta P^{2}}$, where $r=\Delta \hat{P}-\Delta P$ are the residual errors associated with the multiple linear regression.

To investigate whether errors were corrected over multiple gait cycles (Fig. 4), we investigated the correlations between successive lateral foot placements for each leg. We computed these correlations between lateral foot placements at time t and those at time t+N, where N is an integer value capturing the lookup horizon. We then obtained the exponential decay capturing the variation of the Pearson coefficient with the lookup horizon. We used bootstrap resampling (n=10,000) to evaluate the parameters of this exponential decay and reported the 90% CI for each of the parameters we studied.

## Supplementary Material

Appendix 01 (PDF)

## Data Availability

Code for the data processing and analyzes have been deposited in GitHub (https://github.com/MIT-MotorControl/FootPlacementControlAcrossSpecies) (112). Previously published data were used for this work (https://doi.org/10.34770/bzkz-j672; https://doi.org/10.5061/dryad.3p9h20r; https://doi.org/10.5061/dryad.5kh00; and https://www.epic.gatech.edu/opensource-biomechanics-camargo-et-al/) (113–116).
