# Supplementary material for: Foot placement control underlies stable locomotion across species
Source: Proc Natl Acad Sci U S A. 2025 Oct 21;122(43):e2413958122. doi: 10.1073/pnas.2413958122 (PMC12582247; doi:10.1073/pnas.2413958122)
Supplement: Supplementary file 1 — Appendix 01 (PDF) [file pnas.2413958122.sapp.pdf]

SUPPLEMENTARY INFORMATION AND FIGURES FOR "FOOT PLACEMENT CONTROL UNDERLIES STABLE LOCOMOTION ACROSS SPECIES"

Antoine De Comite[1] and Nidhi Seethapathi[1,2,3,*]

[1]McGovern Institute for Brain Research, MIT, Cambridge MA 02139, USA
[2]Department of Brain and Cognitive Sciences, MIT, Cambridge MA 02139, USA
[3]Department of Electrical Engineering and Computer Science, MIT, Cambridge MA 02139, USA
[*]Correspondence should be addressed to N. Seethapathi: nidhise@mit.edu

S1 Methodological details for calculating gait and phase events

Velocity-based contact detection method

In the main paper, we detected the timing of contact initiation and termination by using the maxima and minima of the fore-aft distance between the feet and the body markers. This method is prone to systematic bias in the estimation of contact timing if there is leg retraction prior to contact initiation. Leg retraction consists of a rearward movement of the leg just before heel strike. In the presence of such leg retraction, our method will detect a maximum when leg retraction starts, which does not necessarily correspond to contact initiation. To validate our contact detection method and guarantee that leg retraction was not systematically biasing our contact detection, we compared our method's prediction to that of another method which also considers the foot velocity for contact detection, seeking to avoid artifacts linked to leg retraction [14]. Briefly, this second method uses a threshold on velocity alongside the maximum distance in the fore-aft direction to identify contact initiation. For each detected maximum of the fore-aft distance (i.e. putative foot strike), we searched the first time at which the foot velocity dropped below a threshold. We observed that the fore-aft and lateral location of individual foot contact detected with both methods, defined as the average position of the foot during the stance phase, were extremely similar (Figure S1) and that the predictions of the body state error-based feedback control module were unaltered when the velocity-based method is used (Figure S2). However, the velocity-based method introduced some noise in the estimation of contact location (as illustrated in Figure S1A), we therefore used the position method in the main manuscript.

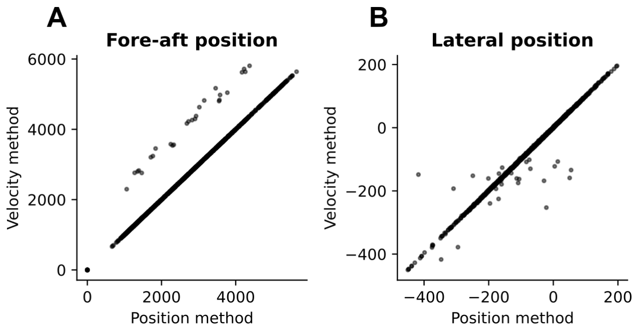

Figure S 1 – Fore-aft (**A**) and lateral (**B**) contact location predictions for the position-only method (horizontal axis) and the velocity-based method (vertical axis) in humans.

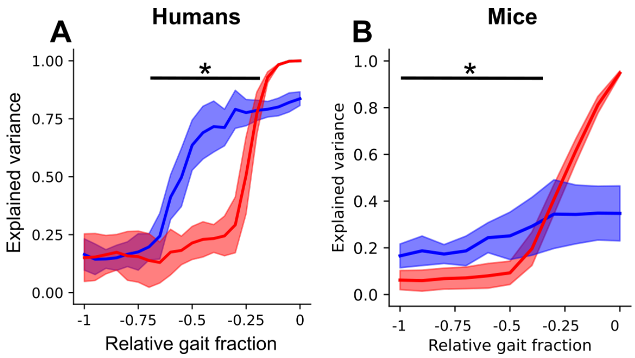

Figure S 2 – Explained variance (R^2, median and interquartile range) obtained for the body state-based (blue) and baseline (red) predictions of the lateral deviations in foot placement for humans (**A**) and mice (**B**) using the velocity-based contact detection method.

Gait segmentation

When investigating the signatures of body state error-dependent foot placement, we used a gait segmentation based on the initiation of foot contact, i.e. touchdown-to-touchdown gait segmentation. To investigate whether this choice of gait segmentation altered our interpretation of the signatures, we conducted the same analyses by using a gait segmentation based on the initiation of the swing phase, i.e. liftoff-to-liftoff gait segmentation. This segmentation based on liftoff reveals qualitatively similar signatures as reported in the main manuscript (Figure S3). We therefore did not observe any effect of the choice of gait events used for segmentation on the finding of the body state error-dependent foot placement control signatures.

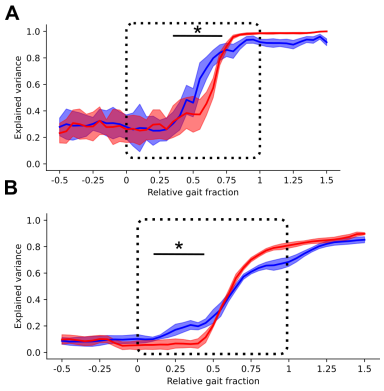

Figure S 3 – Explained variance (R^2, median and interquartile range) obtained for the body state-based (blue) and baseline (red) predictions of lateral deviations in foot placement in humans (**A**) and flies (**B**) for a liftoff-to-liftoff gait segmentation. The black dashed box represents the phase window shown in Figure 3 of the main manuscript. The stars indicate the phase domain where the body state-based model outperformed the baseline (2 samples Kolmogorov-Smirnov tests).

Phase estimation

We compared the method we used to estimate the relative gait fraction to another method that estimates the locomotion phase with a dedicated algorithm [11]. Briefly, this method first computes the Hilbert transform of the fore-aft distance of each individual foot. Then, extracts the *protophase* of each leg as the complex argument of this Hilbert transform. Finally, the individual phases are combined in a single phase estimate using dimensionality reduction techniques. We compared the phase estimations obtained with this method (*oscillators-based method*) to those obtained by linearly interpolating the time vector between two successive contacts of the same leg (*time-based method*) and did not observe any systematic differences across species (Figure S4A-C).

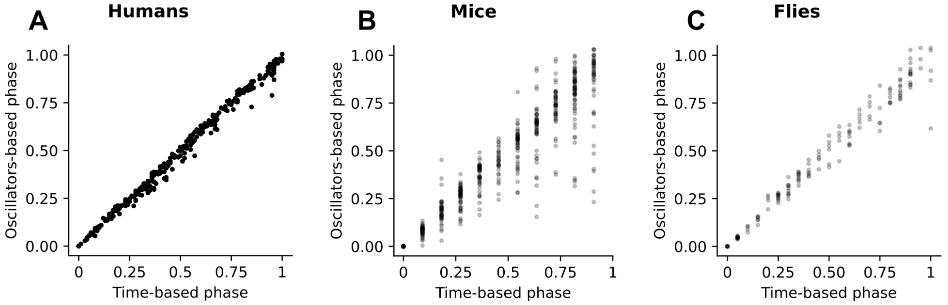

Figure S 4 – Comparison of the relative gait fraction obtained by the time-based method (horizontal axis) and the oscillators-based method (vertical axis) for humans (**A**), mice (**B**), and flies (**C**).

S2 Analyzing the relationship between foot placement and future error reduction

We analyzed the potential stabilizing effects of the foot placements by correlating them to the error reduction on the next step. We defined the error reduction as the difference between the average body state errors during the half gait cycle following contact and the average body state error during the half gait cycle preceding contact. In the main manuscript, we reported the statistics associated with the linear correlations between lateral foot placement and lateral error reduction (Figure 5D-F). The fore-aft deviations in foot placement, on the other hand, were not correlated with fore-aft error reduction (Figure 5A-C). This finding is consistent with our conclusion that there is body state-dependent error correction in the lateral but not in the fore-aft direction.

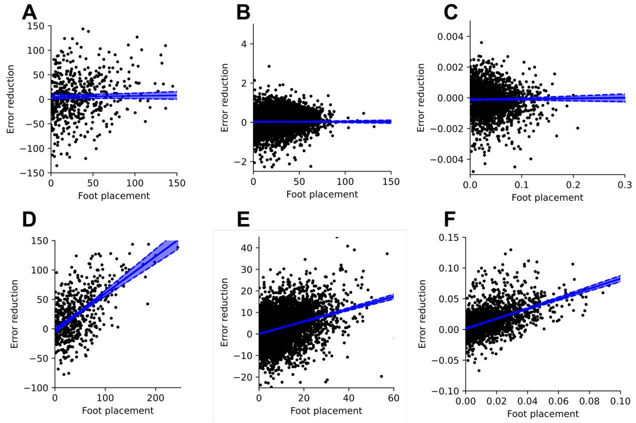

Figure S 5 – Error reduction as a function of foot placement deviation for the fore-aft (top row) and lateral (bottom row) directions for humans (leftmost column), mice (central column), and flies (rightmost column). The associated linear regression and their corresponding 95% confidence intervals are represented in blue full lines and shaded areas, respectively.

S3 Quasi-experimental causal inference of foot placement control signatures

Scientific domains like economics [8], neuroscience and behavior [9] use quasi-experimental methods for causal inference using observational data. Inspired by the *differences-in-differences* method, we quantify the effect of body state-dependent foot placement control during the previous step on the body state error reduction on the next step. For a given small range of positive body-state errors on the previous step, we defined the treatment and the no-treatment groups as the trials with positive and negative body state-dependent foot placement errors respectively. We then compared the error reduction associated with these two groups and reported those for which the distribution of error reduction is significantly greater than zero, corresponding to a reduction in body state-error associated with that specific contact. We found a significant effect in the treatment group for all three species (Figure 6A-C).

The main challenge in extending these quasi-experimental approaches to a new domain such as ours is determining what the treatment variable is. In the case presented here, we considered the presence of body state-dependent deviations in foot placement as treatment and performed the subsequent analyses accordingly. In future extensions of this work that

include mechanical or neural perturbations, these interventions could be considered treatment variables, enabling a quantitative test of the putative causal relationship between body state errors and foot placement corrections.

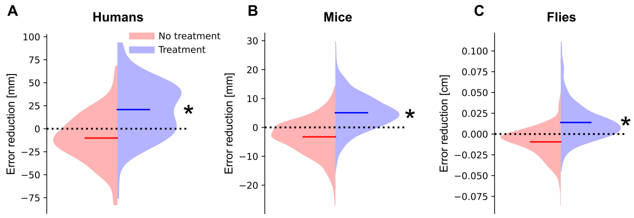

Figure S 6 – Distributions of error-reduction for the treatment (blue) and no-treatment (red) groups for humans **A**, mice **B**, and flies **C**. The dashed black horizontal lines represent an absence of error reduction, positive and negative values respectively capture reduction and increase in body state errors. Significance is reported for the distributions statistically larger than zero. *: $p < 0.05$

S4 Multisensory correction of state drift during locomotion through foot placement control

Drift can be generally defined as the persistent accumulation of errors in the body states (including positions and velocities) relative to a desired trajectory, manifesting as an unintended change in body state over time. Our findings, which identify signatures of error-dependent foot placement control, can be taken to imply that appropriate foot placement control gains contribute to preventing such drift in the body state. While the literature primarily highlights the role of vision in correcting drift across species [4, 7, 10, 13], we propose that the foot placement control gains and their influence on drift can rely on other sensory inputs like proprioception or vestibular feedback [2, 5, 1, 12] and their interactions with biomechanics. In the healthy populations we analyze, properly tuned foot placement control gains likely prevent drift by averting the accumulation of errors. However, if any sensory contribution to foot placement control were incorrectly tuned, drift could theoretically occur as errors build up. Thus, determining the contributions of each sensory modality to overall body state error-dependent foot placement control is an open question [3]. Our data-driven signatures can be combined with studies involving ablations [12] or optogenetic manipulations [6] of sensory inputs to clarify the possible multisensory contributions to preventing the accumulation of body state errors. Such research combining systems neuroscience with our behavioral signatures could discover whether each sensory modality handles body state errors of a specific range of control amplitudes (see Figure 3 in the main manuscript) or timescales (see Figure 4 in the main manuscript). Importantly, such studies would need to focus on immediate behavioral changes following the sensory manipulation to avoid capturing compensatory adaptation mechanisms instead of focusing on identifying the multisensory basis of natural foot placement control.

References

[1] E Azim and Seki K. Gain control in the sensorimotor system. *Current opinion in physiology*, 8:177–187, 2019.

[2] M Bove, G Courtine, and M Schieppati. Neck muscle vibration and spatial orientation during stepping in place in humans. *Journal of Neurophysiology*, 88:2232–2241, 2001.

[3] S M Bruijn and J H van Dieën. Control of human gait stability through foot placement. *Journal of the Royal Society Interface*, 15, 2018.

[4] M E Chiappe. Circuits for self-motion estimation and walking control in drosophila. *Current Opinion in Neurobiology*, 81:102748, 2023.

[5] C J Dakin, J T Inglis, R Chua, and J-S Blouin. Muscle-specific modulation of vestibular reflexes with increased locomotor velocity and cadence. *Journal of neurophysiology*, 110(1):86–94, 2013.

[6] Maxime Fougère, Cornelis Immanuel van der Zouwen, Joël Boutin, Kloé Neszvecsko, Philippe Sarret, and Dimitri Ryczko. Optogenetic stimulation of glutamatergic neurons in the cuneiform nucleus controls locomotion in a mouse model of parkinson's disease. *Proceedings of the National Academy of Sciences*, 118(43):e2110934118, 2021.

[7] T Fujiwara, M Brotas, and M E Chiappe. Walking strides direct rapid and flexible recruitment of visual circuits for course control in drosophila. *Neuron*, 110:2124–2138, 2022.

[8] M Lemieux, N Josset, M Roussel, S Couraud, and F Bretzner. Speed-dependent modulation of the locomotor behavior in adult mice reveals attractor and transitional gaits. *Frontiers in Neuroscience*, 10, 2016.

[9] I E Marinescu, P N Lawlor, and K P Kording. Quasi-experimental causality in neuroscience and behavioural research. *Nature Human Behaviour*, 2:891–898, 2018.

[10] T Proko, M Schubert, and W Berger. Visual influence of human locomotion modulation to changes in optic flow. *Experimental Brain Research*, 114:63–70, 1997.

[11] S Revzen and J M Guckenheimer. Estimating the phase of synchronized oscillators. *Physical Review E*, 78:051907, 2008.

[12] L Ruder, A Takeoka, and S Arber. Long-distance spinal neurons ensure quadrupedal locomotor stability. *Neuron*, 92:1063–1078, 2016.

[13] A B Saleem, A Ayaz, K J Jeffery, K D Harris, and M Carandini. Integration of visual motion and locomotion in mouse visual cortex. *Nature Neuroscience*, 16:1864–1869, 2013.

[14] J A Zeni Jr, J G Richards, and J S Higginson. Two simple methods for determining gait events during treadmill and overground walking using kinematic data. *Gait & Posture*, 27:710–714, 2008.
